# Highly conserved antigenic epitope regions of hemagglutinin and neuraminidase genes between 2009 H1N1 and seasonal H1N1 influenza: vaccine considerations

**DOI:** 10.1186/1479-5876-11-47

**Published:** 2013-02-22

**Authors:** Ping Huang, Shouyi Yu, Changyou Wu, Lijun Liang

**Affiliations:** 1 Key Laboratory for Emergency Pathogen Detection, Guangdong Provincial Center for Disease Control and Prevention, Guangzhou, 511430, China; 2 Department of Epidemiology, School of Public Health and Tropic Medicine, Southern Medical University, Guangzhou, 510515, China; 3 Department of Epidemiology, School of Public Health, Sun Yat-Sen University, Guangzhou, 510080, China; 4 Institute of Immunology, Zhongshan School of Medicine, Sun Yat-Sen University, Guangzhou, 510080, China

**Keywords:** Influenza, H1N1, Epitope, Vaccine, Immunoinformatic

## Abstract

An immunoinformatics study was conducted to determine the highly conserved antigenic epitope regions of hemagglutinin (HA) and neuraminidase (NA) genes in the humoral immunity and CD4+ and CD8+ T cellular immunity between 2009 pandemic H1N1 (pH1N1) and seasonal H1N1 (sH1N1) viruses. It was found that in sH1N1 viruses, 29 epitope regions of HA genes and 8 epitope regions of NA genes which had been experimentally identified, were highly conserved (97.1-100.0%) in the corresponding genes and predictive epitopes of the pH1N1 viruses. The results suggested that highly conserved antigenic epitope regions might act as the basis of common antigenic vaccines against pH1N1 and sH1N1 viruses.

## Introduction

On April 15 2009, a novel pandemic influenza A (pH1N1) virus was identified from specimens obtained from influenza patients in the United States, subsequently, large numbers of confirmed cases of human pH1N1 have been identified worldwide [1]. The pH1N1 virus contains a combination of gene segments that has not previously been reported in swine or human influenza viruses either in the USA or elsewhere [2]. Viruses with a NA gene segment were originally derived from a wholly avian influenza virus, which entered the Eurasian swine population in 1979. It continued to circulate throughout Eurasia [3], however, it had not been previously reported outside this region. The HA gene segment was part of the classical swine lineage with the virus infecting pigs around 1918 and subsequently circulated in classical swine viruses and triple reassortant swine viruses [4].

Influenza pandemics occur when human have no immunity against a particular influenza virus, in this case, containing both HA and NA genes that evolve to be efficiently transmitted from human-to-human. The genomes of the last three pandemic influenza viruses (1918 H1N1, 1957 H2N2 and 1968 H3N2) all originated either wholly or partly from non-human reservoirs, with the HA genes of all pandemic viruses ultimately originating from avian influenza viruses [2]. The pH1N1 virus induced most neutralizing antibodies, which were broadly cross-reactive against epitopes in the hemagglutinin (HA) stalk and head domain of multiple influenza strains, including the seasonal H1N1 virus (sH1N1) [5]. Based on the above observations, it was postulated that the plasmablasts that produced these broadly neutralizing antibodies were predominantly derived from activated memory B cells specific for epitopes conserved in some influenza strains. As immunity against influenza viruses involved not only B cell activation, but also T cell recognition and presentation, the T cell subgroups undoubtedly play an important role. By performing large-scale major histocompatibility complex (MHC) II analyses on HA proteins, the degree of T-cell cross-reactivity between sH1N1 from 1968 to 2009 and pH1N1 strains was investigated. The T-cell cross-reactivity was estimated at 52% between sH1N1 and pH1N1 [6]. From biological studies using peripheral blood mononuclear cell (PBMC) from human donors not previously exposed to the pandemic virus, pre-existing CD4+ T cells can elicit cross-reactive effector responses against the pandemic H1N1 virus. Computational tools were 80–90% accurate in predicting CD4+ T cell epitopes and their HLA-DRB1 -dependent response profiles in donors selected at random for human leukocyte antigen (HLA) haplotype [7]. HLA, also known as human MHC, is classically divided MHC I, II, III with human immunity against influenza involving MHC I and MHC II alleles in influenza. In this study, the highly conserved antigenic epitopes sequences and the locations of HA and NA proteins, including B-cell epitopes, MHC I and MHC II epitopes between pH1N1 and sH1N1 viruses, based on immunoinformatics, were analyzed. There were compared with epitopes in the Immune Epitope Database (IEDB) which contains experimentally identified epitopes in sH1N1 HA proteins, which may be helpful in design of common antigens of pH1N1 influenza and sH1N1 viruses.

## Methods

### Gene sequences sequenced and downloaded

The HA and NA gene sequences of nine pH1N1 viruses isolated from Guangdong, China, were sequenced (GenBank accession numbers GU471691–GU471695 and GU562466– GU562469 for NA and CY120915–CY120924 and CY120952 for HA), and the global corresponding genes (59 isolates) were obtained from GenBank. The 1495 HA and 2005 NA gene sequences of sH1N1 influenza isolated between 1998 and 2008 were downloaded from GenBank.

### Prediction of B-cell epitopes, MHC I Epitopes and MHC II Epitopes

The B-cell epitopes containing at least 10 amino acids were predicted by ABCpred with a threshold values of 0.51 [8]. The MHC I molecules predicted by both BIMAS (explicit number = 100) and by SYFPEITHI (score ≥10.0) [9,10] were conducted, respectively, with the predictive epitopes containing at least 10 amino acids. The same epitope sequences predicted by both BIMAS and SYFPEITHI were determined. The MHC II molecules predicted by SYFPEITHI (score ≥10.0) with epitopes comprised of at least 10 amino acids were done [10].

### ***Epitopes in*** IEDB

The HA and NA gene sequences of sH1N1 viruses isolated between 1998 and 2008 and pH1N1 viruses isolated in 2009 were aligned by Clustal-W of MEGA 5.05 [11]. The amino acid sequences were analyzed and the conserved ratios were calculated. The previously experimentally identified epitopes of HA and NA proteins in sH1N1 proteins in IEDB were selected [12].

### Validation of conversed epitopes

The predictive epitope sequences of pH1N1 genes were compared with the corresponding genes of sH1N1 and those with < 90% of conserved regions were rejected. The remaining predictive epitope sequences of the pH1N1 genes were compared with epitopes in the IEDB with only those epitopes of sH1N1 genes with conserved regions of > 90% being selected. Finally, the only epitopes with > 95% conserved in IEDB of sH1N1 genes as well as the predictive epitope of pH1N1 was selected.

### Homology Modeling

The homology model was generated using the SWISS-MODEL homology-modeling server [13] and decorated using the Cimera [14]. Some epitope regions were labeled in the three- dimensional (3D) structure.

## Results

### Prediction of B-cell epitopes, MHC I Epitopes and MHC II Epitopes

The HA and NA proteins of A/Guangdong/801/2009 (pH1N1) contained 567 amino acids coded by 1701 nucleotides and 470 amino acids coded by 1410 nucleotides, respectively; each having the identical gene lengths of the sH1N1 virus. The HA and NA proteins of 68 pH1N1 viruses were aligned to illustrated the variations in the proteins.

The B-cell epitopes predicted by ABCpred with a threshold values of 0.51 were 330 in HA and 278 in NA. The MHC I molecules in HLA-A1, HLA-A0201, HLA-A3 and HLA-A1101 of HA and NA proteins predicted by BIMAS (number of top-scoring = 100) were 400 in HA and 400 in NA, meanwhile those in HLA-A*01, HLA-A*0201, HLA- A*03 and HLA-A*1101 predicted by SYFPEITHI were 400 in HA and 400 in NA. The MHC II molecules in HLA-DRB1*0101, HLA-DRB1*0301, HLA-DRB1* 0401 HLA- DRB1*0701, HLA-DRB1*1101, and HLA-DRB1*1501 of HA and NA proteins predicted by SYFPEITHI were 1423 in HA and 1146 in NA.

### Gene alignment and IEDB epitope

The 1432 (95.8% of 1495) proteins of sH1N1 HA and the 1928 (96.2% of 2005) proteins of sH1N1 NA were aligned after the reduplications in downloaded sequences were discharged. According to the above predictive B-cell epitope, MHC I molecules and MHC II molecules of pH1N1, the conserved ratio of each sH1N1 epitope sequence was acquired. Forty-six epitope sequences of sH1N1 HA proteins were searched in IEDB (291 epitopes of sH1N1 HA in IEDB) for those which had 90%-100% of conserved ratios and twenty-one epitope sequences of NA proteins were done (80 epitopes of sH1N1 NA in IEDB). The epitopes downloaded in IEDB mixed both B-cell epitopes and T-cell epitopes.

### Conserved epitope

There were twenty-nine conserved epitope sequences in HA proteins with 97.1%-100% of conserved ratios between sH1N1 and pH1N1. Epitope SVIEKMNTQFTAV (IEDB No.80042, aa398-410) was overlapped with epitope SVIEKMNTQFTAVGKE (IEDB No.127161, aa398-413), shown in Table 1. There were eight conserved epitope sequences in NA proteins with conserved ratios of 99.3-99.9% between sH1N1 and pH1N1, shown in Table 2.

**Table 1:** Conservancy of hemagglutinin (HA) epitopes of sH1N1 and pH1N1 influenza viruses

**HA sequence epitope**^**a**^	**Number (%) of study years conserved**^**b**^	**Epitope id in IEDB**	**Possible B-cell epitope**^**c**^	**Possible MHC I allelesd**	**Possible MHC II allelese**
IGYHANNSTDTVDTVLEK	99.9	95458	IGYHANNSTD(22–31)	A*01,A*1101	DRB1*0101,DRB1*0301,DRB1*0401,
(22–39)			NSTDTVDTVL(28–37)		DRB1*0701, DRB1*1501
HANNSTDTVDTVLEKNV	99.9	128846	NSTDTVDTVL(28–37)	A*01,A*1101	DRB1*0101,DRB1*0301,DRB1*0401,
(25–41)					
STDTVDTVLEKNVTVTHS	99.9	95880	STDTVDTVLEKNVTVTHS	A*01,A*0201,	DRB1*0101,DRB1*0301,DRB1*0401,
(29–46)			(29–46)	A*03,A*1101	DRB1*0701, DRB1*1101,DRB1*1501
DTVDTVLEKNVTVTHSV	99.8	128470	NSTDTVDTVL(28–37)	A*01,A*0201,	DRB1*0101,DRB1*0301,DRB1*0401,
(31–47)			DTVLEKNVTVTH(34–45)	A*03,A*1101	DRB1*0701, DRB1*1101,DRB1*1501
DYEELREQLSSVSSFER	99.8	128481	YEELREQLSSVSSF	A*01,A*0201	DRB1*0101,DRB1*0301,DRB1*0401,
(114–130)			(115–128)	A*03,A*1101	DRB1*0701, DRB1*1101,DRB1*1501
EQLSSVSSFERFE	99.8	113375	*	A*0201,A*03,	DRB1*0701
EQLSSVSSFERFEIFPK	99.9	128569	QLSSVSSFERFEIF	A*0201,A*03,	DRB1*0101,DRB1*0301,DRB1*0401,
(120–136)			(121–134)	A*1101	DRB1*0701, DRB1*1101,DRB1*1501
IQSRGLFGAIAGFIEGG	99.9	128979	SRGLFGAIAG	A*0201,A*03,	DRB1*0101,DRB1*0301,DRB1*0401,
(341–357)			(343–352)	A*1101	DRB1*0701,DRB1*1501
FGAIAGFIEGGWTGMVD	99.2	128623	AIAGFIEGGW	A*1101	DRB1*0101,DRB1*0301,DRB1*0401,
(347–363)			(349–358)	DRB1*0701	
FIEGGWTGMVDGWYGYH	99.2	128629	EGGWTGMVDGWY(355–366)	A*1101	DRB1*0101,DRB1*0401,DRB1*0701,
(353–369)			EGGWTGMVDGWYGY(355–368)		DRB1*1501
TGMVDGWYGYHHQNEQG	99.2	130077	TGMVDGWYGYHH(359–370)	A*1101	DRB1*0101,DRB1*0301,DRB1*0401,
(359–375)					DRB1*0701,DRB1*1101,DRB1*1501
TGMVDGWYGYHHQNEQGS	99.2	95905	TGMVDGWYGYHH(359–370)	A*1101	DRB1*0101,DRB1*0301,DRB1*0401,
(359–376)					DRB1*0701,DRB1*1101,DRB1*1501
WYGYHHQNEQGSGYAAD	100.0	130354	YGYHHQNEQGSGYA	A*1101	DRB1*0101,DRB1*0401,DRB1*0701,
(365–381)			(366–379)	DRB1*1101	
TNKVNSVIEKMNTQFTA	99.6	130108	TNKVNSVIEKMNTQFTAV	A*0201,A*1101	DRB1*0101,DRB1*0301,DRB1*0401,
(393–409)			(393–410)		DRB1*0701,DRB1*1101,DRB1*1501
NKVNSVIEKMNTQFTAVG	99.6	95623	*	A*1101	DRB1*0101,DRB1*0301,DRB1*0401,
(394–411)					DRB1*0701,DRB1*1101,DRB1*1501
SVIEKMNTQFTAV(398–410)	99.5	80042	*	A*0201,A*1101	DRB1*0101,DRB1*0401,DRB1*1501
SVIEKMNTQFTAVGKE(398–413)	99.	127161			
VIEKMNTQFTAVGKEFN	99.4	130227	KMNTQFTAVGKEFN(402-415)	A*0201,A*1101	DRB1*0101,DRB1*0301,DRB1*0401
(399–415)					DRB1*0701,DRB1*1101,DRB1*1501
EKMNTQFTAVGKE(401–413)	99.9	7980	*	A*0201,A*03, A*1101	DRB1*0101,DRB1*0401,DRB1*1501
NLNKKVDDGFLDIWTYN	97.4	129494	*	A*01,A*0201,	DRB1*0101,DRB1*0301,DRB1*0401,
(423–439)				A*03,A*1101	DRB1*0701,DRB1*1501
DDGFLDIWTYNAELLVL	97.5	128403	DDGFLDIWTYNAEL(429–442)	A*01,A*0201,	DRB1*0101,DRB1*0301,DRB1*0401
(429–445)				A*03,A*1101	DRB1*0701,DRB1*1101,DRB1*1501
DGFLDIWTYNAELLV	97.1	113324	*	A*1101	DRB1*0101,DRB1*0301,DRB1*0401
(430–444)					DRB1*0701,DRB1*1101,DRB1*1501
IWTYNAELLVLLENERT	99.7	129015	WTYNAELLVLLENE(436–449)	A*01,A*0201,	DRB1*0101,DRB1*0301,DRB1*0401
(435–451)				A*03,A*1101	DRB1*0701,DRB1*1101,DRB1*1501
WTYNAELLVLLENERTLD	100.0	96007	WTYNAELLVLLENE(436–449)	A*01,A*0201,	DRB1*0101,DRB1*0301,DRB1*0401
(436–453)				A*03,A*1101	DRB1*0701,DRB1*1101,DRB1*1501
ELLVLLENERTLD 99.9		79809	*	A*0201,A*1101	DRB1*0101,DRB1*0301,DRB1*0401,
(441–453)					DRB1*0701,DRB1*1501
LKNNAKEIGNGCFEFYH	99.9	129255	KNNAKEIGNGCFEF(471–484)	A*01,A*03,	DRB1*0101,DRB1*0301,DRB1*0401,
(470–486)				A*1101	DRB1*0701
MESVKNGTYDYPKYSEE	99.8	129388	*	A*01,A*0201,	DRB1*0101,DRB1*0301,DRB1*0701,
(493–509)				A*03,A*1101	DRB1*1101,DRB1*1501
SLGAISFWMCSNGSLQ	99.7	129938	*	A*0201,A*03,	DRB1*0301,DRB1*0401, DRB1*1101,
(546–561)				A*1101	DRB1*1501
SFWMCSNGSLQCRICI	99.3	129890	*	A*1101	DRB1*0101,DRB1*0301,DRB1*0401,
(551–566)					DRB1*0701,DRB1*1501

a. Epitopes in IEDB were experimentally identified epitopes in sH1N1 HA genes. b. The conservancy ratio of 1432 genes of sH1N1 HA sequences, whose strains isolated from 1968 to 2008. c. The B-cell epitopes of pH1N1 HA genes are predicted by ABCpred in 10 mer or more, respectively. d. MHC I alleles of 10 mer and more of pH1N1 HA genes are Predicted by BIMAS (explicit number = 100) and SYFPEITHI (score ≥10.0), respectively. The same epitope sequences predicted by both BIMAS and SYFPEITHI were determined. e. MHC II alleles of 10 mer and more of pH1N1 HA genes are predicted by SYFPEITHI (score ≥10.0), respectively.

**Table 2:** Conservancy of neuraminidase (NA) epitopes of sH1N1 and pH1N1 influenza viruses

**NA sequence epitope**^**a**^	**Number (%) of study years conserved**^**b**^	**Epitope id in IEDB**	**Possible B-cell epitope**^**c**^	**Possible MHC I alleles**^**d**^	**Possible MHC II alleles**^**e**^
KDNSIRIGSKGDVFVIR	99.8	129048	SIRIGSKGDV(105–114)	A*0201,A*03,	DRB1*0101,DRB1*0301,DRB1*0401
(102–118)			DNSIRIGSKGDVFV(103–116)	A*1101	DRB1*0701,DRB1*1101,DRB1*1501
IGSKGDVFVIREPFIS	99.8	128924	GDVFVIREPF(112–121)	A*0201,A*03, A*1101	DRB1*1101,DRB1*1501,
RTFFLTQGALLNDKHSN	99.5	127810	FLTQGALLND(133–142)	A*01,A*03,	DRB1*0101,DRB1*0301,DRB1*0401
(130–146)			FFLTQGALLNDKHS(132–145)	A*1101	DRB1*0701,DRB1*1101,DRB1*1501
ISGPDNGAVAVLKYNGI	99.5	128994	ISGPDNGAVAVLKY(195–208)	A*01,A*0201	DRB1*0301,DRB1*0401,DRB1*1501
(195–211)			GPDNGAVAVL(197–206)		
VCRDNWHGSNRPWVSFN	99.9	130190	CRDNWHGSNRPW(292–303)	A*01	DRB1*0101,DRB1*0401,DRB1*0701,
(291–307)			RDNWHGSNRPWVSF(293–306)		DRB1*1101
WSGYSGSFVQHPELTGL	99.9	130342	*	A*0201,A*03,	DRB1*0101,DRB1*0401,DRB1*0701,
(399–415)				A*1101	DRB1*1101,DRB1*1501,
SFVQHPELTGLDCIRP	99.2	129889	*	A*01, A*0201,	DRB1*0101,DRB1*0401,DRB1*1501
(405–420)				A*03, A*1101	
PELTGLDCIRPCFWVEL	99.3	129560	*	A*01,A*0201,	DRB1*0101,DRB1*0301,DRB1*0401
(410–426)				A*03,A*1101	DRB1*0701,DRB1*1101,DRB1*1501

a. Epitopes in IEDB were experimentally identified epitopes in sH1N1 NA genes. b. The conservancy ratio of 1928 genes of sH1N1 NA sequences, whose strains isolated from 1968 to 2008. c. The B-cell epitopes of pH1N1 NA genes are predicted by ABCpred, respectively. d. MHC I alleles of 10 mer and more of pH1N1 NA genes are Predicted by BIMAS (explicit number = 100) and SYFPEITHI (score ≥10.0), respectively. The same epitope sequences predicted by both BIMAS and SYFPEITHI were determined. e. MHC II alleles of 10 mer and more of pH1N1 NA genes are predicted by SYFPEITHI (score ≥10.0), respectively.

Only 62.1% (18/29) of predictive B-cell epitopes of pH1N1 HA proteins overlapped the conserved epitope sequences in sH1N1 HA proteins. The predictive sequences of MHC I molecules and MHC II molecules of pH1N1 covered almost all the conserved epitope sequences in sH1N1 HA proteins, but the positive ratios in HLA-A*0101, A*0201, A*03 and A*1101 were 37.9% (11/29), 62.1% (18/29), 48.3% (14/29) and 100% (29/29), respectively. Only 37.5% (3/8) of predictive B-cell epitopes of pH1N1 NA proteins overlapped the conserved epitope sequences in sH1N1 NA proteins. The predictive sequences of MHC I molecules and MHC II molecules of pH1N1 covered almost all the conserved epitope sequences in sH1N1 NA proteins, but the positive ratios in HLA-A*01, A*0201, A*03 and A*1101 were 62.5% (5/8), 75.0% (6/8), 50.0% (4/8) and 75.0% (6/8), respectively.

The predictive epitopes of MHC I and MHC II molecules of pH1N1 covered almost all the conserved epitope sequences in sH1N1 HA proteins, with the positive ratios in HLA-DRB1*0101, DRB1*0301, DRB1*0401, DRB1*0701, DRB1*1101 and DRB1*1501 being 86.2% (25/29), 79.3% (23/29), 93.1% (27/29), 82.8% (24/29), 55.2% (16/29) and 82.8% (24/29), respectively; and those in six HLA-DRB1 alleles were 75.0% (6/8), 50.0% (4/8), 87.5% (7/8), 62.5% (5/8), 75.0% (6/8) and 87.5% (7/8), respectively.

### 3D structure

**Figure 1:**
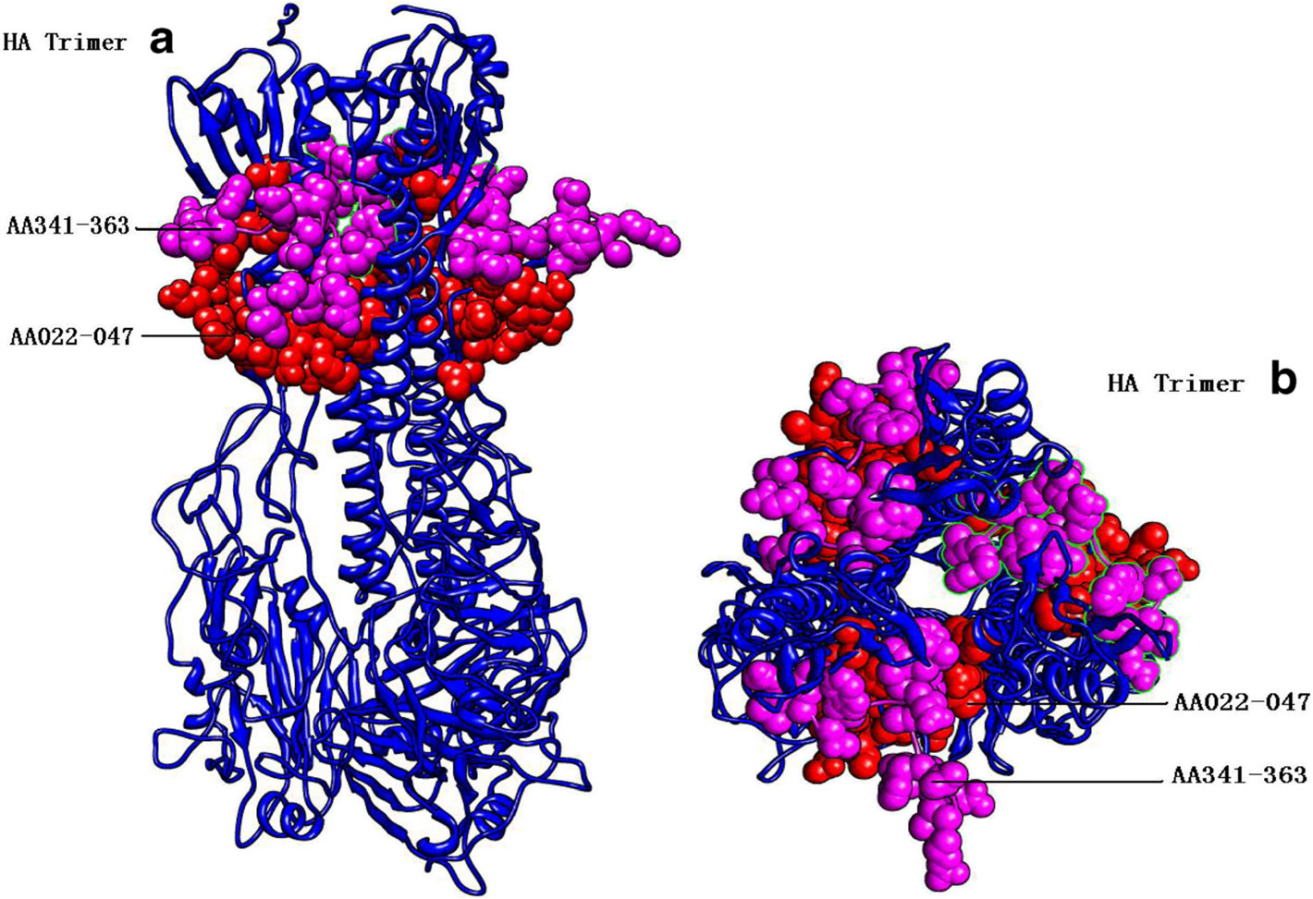
**HA tetramer decorated with two epitope regions. a**. Side view; **b**. Vertical view. AA22-47 and AA341-363 are IGYHANNSTDTVDTVLEKNVTVTHSV in red and IQSRGLFGAIAGFIEGGWTGMVD in magenta.

The H1 hemagglutinin trimer of strain A/801/2009 was obtained using SWISS-MODEL. The 64.2% identity of 2wr0B in the Protein Data Bank (PDB) spanned amino acid residues from 18 to 511 with 2.45 Å X-ray resolution. The epitope region aa22-47 of the pH1N1 HA protein referred to that of the sH1N1 HA protein in IEDB (No. 95458, 95880, 128470 and 128846) and the epitope region aa341-363 referred to that of the sH1N1 HA protein in IEDB (No.128623 and 128979), whose atoms/bonds structures are shown in Figure 1, and were positively predicted by all three methods (the B-cell epitope ABCpred, the MHC I molecule BIMAS and SYFPEITHI and the MHC II molecule SYFPEITHI. Both epitope regions were neighboring in close proximity in three-dimensional structure although the sequence positions spanned 294 amino acids, as shown in Figure 1.

**Figure 2:**
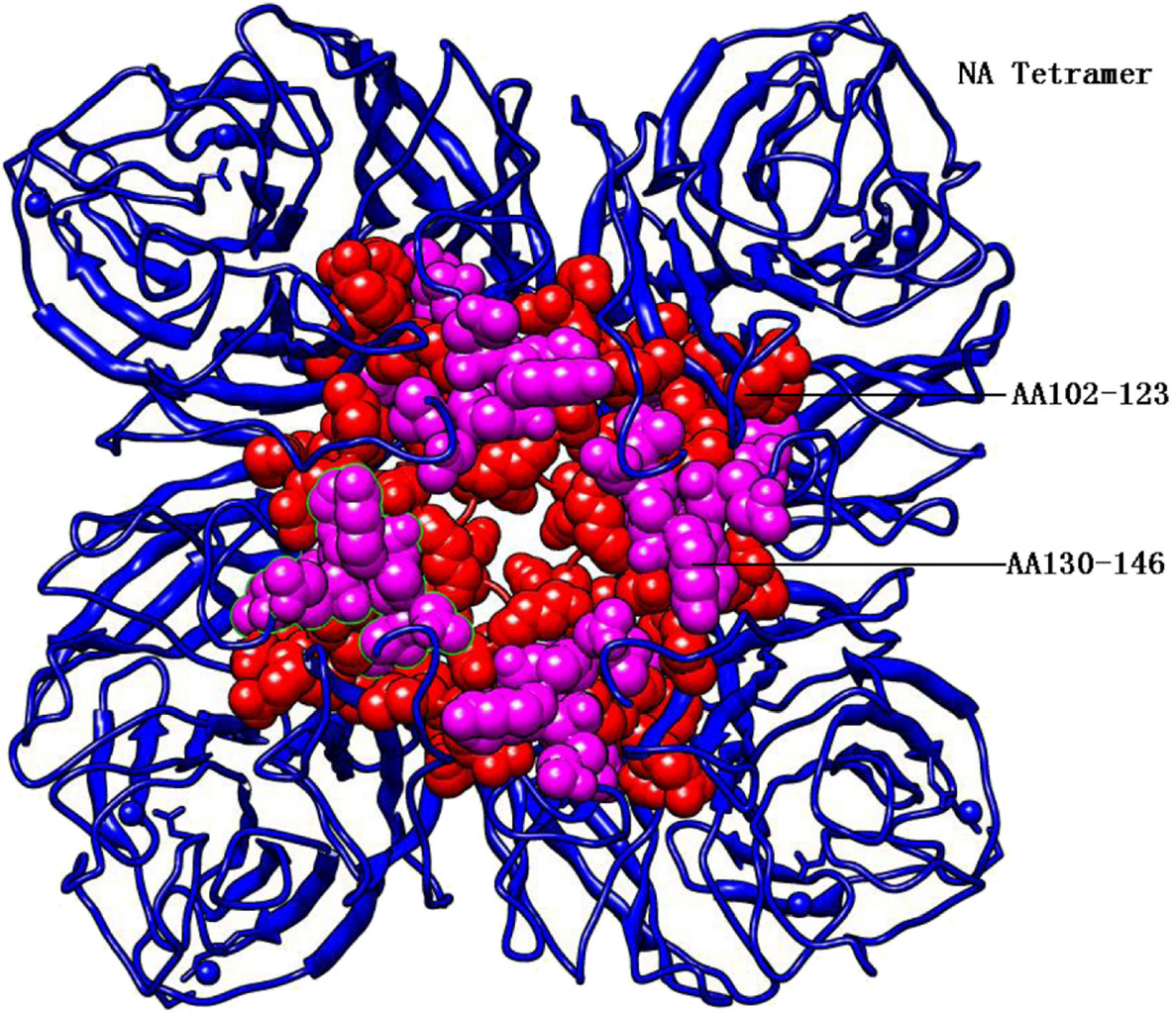
**NA tetramer decorated with two epitope regions.** AA102-123 and AA 130–146 are KDNSIRIGSKGDVFVIREPFIS in red and RTFFLTQGALLNDKHSN in magenta.

The N1 neuraminidase tetramer of strain A/Guangdong/801/2009 was obtained using SWISS- MODEL. The model 3ti4B in the Protein Data Bank (PDB) had the highest sequence homology (99.5% identity) at amino acid residues from 82 to 468 with 1.60 Å X-ray resolution, as shown in Figure 2. There were the epitope region aa102-123 of pH1N1 HA proteins referred to that of sH1N1 HA proteins in IEDB (No.128924 and 129048) and the epitope region aa130-146 of pH1N1 HA proteins referred to that of sH1N1 HA proteins in IEDB (No.127810), whose atoms/bonds structures were shown in Figure 2, and were positively predicted by the three methods. Both epitope regions were close in three-dimensional structure and the sequence positions spanned seven amino acids.

## Discussion

The antigenic epitope analysis performed in this study suggested the presence of pre-existing cross-reactive humoral immunity, CD4+ and CD8+ T cellular immunity to pH1N1 in the human population from the conserved regions of HA and NA proteins. Although the sH1N1 virus emerged since the 1918 pandemic, it continues to circulate among the human population today, and might be the basis of pre-existing cross-reactive immunity [7,15,16]. However, such pre-existing T cell immune responses do not prevent infection once a person has been exposed to a virus [17], although T cell immunity could contribute to the viral clearance of infected cells (target cells), resulting in a reduction in disease severity, and furthermore, may indirectly reduce person-to-person spread [15,18].

Twenty-nine epitope regions of HA genes and eight epitope regions of NA genes which had been experimentally identified in sH1N1 viruses, were highly conserved (97.1-100.0%) in the corresponding genes and predictive epitopes of the pH1N1 viruses. Due to focus on the conserved epitope sequences in this study, the properties of IEDB epitopes (MHC binding, cellular assay, etc.) were excluded. Immune antigens of pathogens mainly involve the B-cell antigen, in addition to MHC I and MHC II molecules. In all conserved epitopes (37/37) in this study, more than half (23/37) of epitopes were B-cell epitopes. Some antigens responded to produce specific antibodies against the antigens. The MHC II molecules were related to CD4+ cell, which involved the antigenic recognition and presentation, were more active than the MHC I molecules related to CD8+ cell in this study.

As common antigens of influenza existed, it is potentially possible for a universal influenza vaccine to be achieved within pathogen genomes based on epitopes [19]. However, epitope selection to design a universal influenza vaccine warrants further research, particularly for the existence of specific B-cell and T-cell epitope repertoires, and also epitope binding to different MHC alleles in the heterogeneous human population. Immunoinformatics is accelerating the development of vaccines comprised of epitope ensembles and the confirmation of these vaccines in human clinical trials will serve to usher in a new era of epitope driven vaccine design [19,20]. Some highly conserved epitopes (including two HA epitope regions and two NA epitope regions in this study) are adjacent in the three-dimensional structure, which suggests the importance of the combination of epitopes when designing vaccine. In summary, these well-characterized epitopes could be combined as potential vaccine candidates and may confer broader cell mediated immunological responses to various subtypes of influenza A viruses. The results in this study suggested that highly conserved antigenic epitope regions might act as the basis of common antigenic vaccines against pH1N1 and sH1N1 viruses.

## Competing interests

The authors declare that they have no competing interests.

## Authors’ contributions

PH: Conceived and designed the study, developed the model and wrote the manuscript. SYY: Designed the study and provided important intellectual concept of the study. CYW: Designed the study and provided support for the study. LJL: Developed the model, wrote the manuscript and provided support for the study. All authors read and approved the final manuscript.
